# Inguinal ovary in adult women-case report and literature review

**DOI:** 10.1186/2193-1801-2-545

**Published:** 2013-10-17

**Authors:** Mette L Josefsson, Surajit Mitra, Sanjay Gupta

**Affiliations:** Department of Obstetrics and Gynaecology, Lister Hospital, Coreys Mill Lane, Stevenage Hertfordshire, SG1 4AB UK; Department of General Surgery, Lister Hospital, Coreys Mill Lane, Stevenage Hertfordshire, SG1 4AB UK

## Abstract

This paper describes a rare finding of an inguinal ovary in an adult woman who presented with pelvic pain. Inguinal ovary may occur if the gubernaculum fails to attach to the uterus or if the canal of Nuck remains open during fetal development.

**Electronic supplementary material:**

The online version of this article (doi:10.1186/2193-1801-2-545) contains supplementary material, which is available to authorized users.

## Introduction

Ovary within the inguinal canal is occasionally seen in infants, but is rare in adult women. This paper describes a woman with an inguinal ovary who presented with pelvic pain. We review the literature and discuss its embryological background.

## Case report

**Figure 1:**
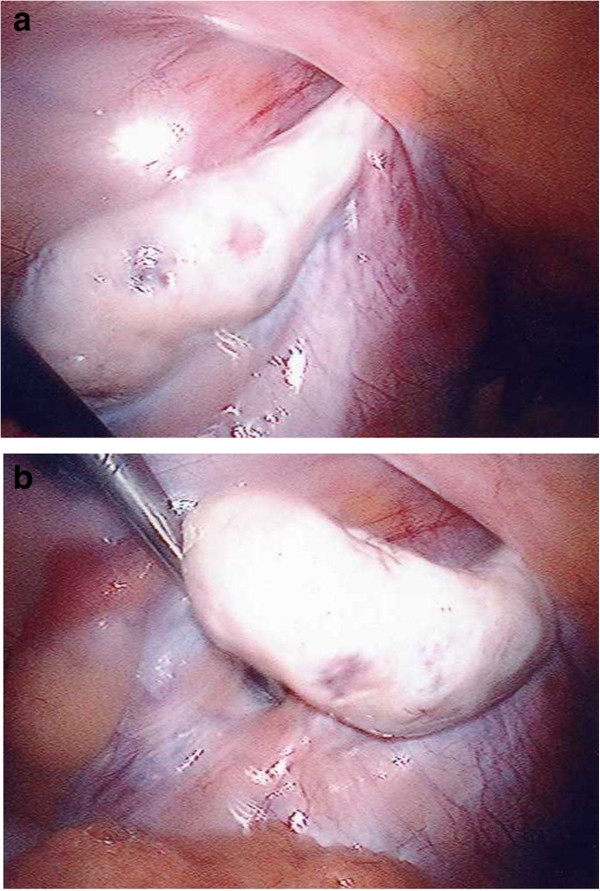
**a and b, Laparoscopic images of left ovary herniating through the left internal inguinal ring.**

A 33 year-old-woman presented with pelvic pain on a background of longstanding left sided iliac fossa pain, which was exaggerated by constipation and movement. Past medical history included depression, scarred left kidney from recurrent urinary tract infections and surgery for urethral diverticulum. Obstetric history included two vaginal deliveries following subfertility treatment. Ultrasound scan demonstrated an unremarkable retroverted uterus and normal renal tract. The right ovary appeared normal however left ovary was not identified. Diagnostic laparoscopy revealed the left ovary herniating through the left internal inguinal ring (Figure 1a-b). The left ovarian and round ligaments were absent and only a small left fallopian tube was seen (Additional file 1: Figure S1). Uterus, and right tube, ovary and round ligament appeared normal. She later underwent laparoscopic mobilization of the ovary and mesh repair of the inguinal canal jointly with the general surgeons (Additional file 2: Figure S2). She recovered well and was discharged home two days later.

### Comment

A literature search revealed twelve case reports in adults. The majority presented with a palpable groin mass and they were diagnosed at time of surgery for suspected bowel hernia (Tagliaabue [2011]; Machado & Machado [2011]; Mandel et al. [2010]; Alzaraa [2011]). One woman presented with pelvic pain and was found on laparoscopy to have a rudimentary uterine horn and ovary herniating through the inguinal canal (Al Omari et al. [2011]). Coexisting mullerian and renal malformations have been described (Alzaraa [2011]; Al Omari et al. [2011]).

Male and female reproductive systems share common steps in early development. Understanding this helps to comprehend the causes of inguinal ovary. The gonads develop from mesothelium on the urogenital ridge. Initially in both male and female, the gonads are situated posterior in the abdominal cavity. After sex differentiation both ovaries and testes descend, but to varying extent. The testes descend to the scrotum via the gubernaculums, a caudal remnant of the mesonephric duct that passes through the abdominal wall to the labioscrotal swelling. As the embryo grows the gubernaculums remains the same size causing the testes to be situated progressively lower down. By the eight month the testes have reached the scrotum and by birth the processus vaginalis at the internal inguinal ring closes.

Contrary to the testes, the ovary descends to a lesser extent because the gubernaculums adheres to the uterus, thus preventing movement. If the gubernaculum fails to attach, the ovary may descend through the inguinal canal. The canal of Nuck is a pouch of peritoneum, analogous to the processus vaginalis in males, which follows the round ligament from the ovary to the labia via the inguinal canal. This canal closes within the first year of life. If it fails to close a hernia may form.

## Conclusion

This paper describes a rare case of inguinal ovary in a woman who presented with pelvic pain. An inguinal ovary may occur if the gubernaculum fails to attach to the uterus in fetal life or if the canal of Nuck remains open after birth.

## Consent

Written informed consent was obtained from the patient for publication of this case report and accompanying images. A copy of the written consent is available for review by the Editor-in-Chief of this journal.

## Electronic supplementary material

Additional file 1: Figure S1: Laparoscopic image of uterus and right ovary. Note the absence of left ovary, left ovarian ligament and left fallopian tube. (JPEG 255 KB)

Additional file 2: Figure S2: Laparoscopic image of left ovary following its removal from the inguinal canal and mesh repair of the hernia. (JPEG 329 KB)

Below are the links to the authors’ original submitted files for images.

Authors’ original file for figure 1
