# Supplementary figures and images for: Inguinal ovary in adult women-case report and literature review
Source: Springerplus. 2013 Oct 17;2(1):545. doi: 10.1186/2193-1801-2-545 (PMC3825092; doi:10.1186/2193-1801-2-545)

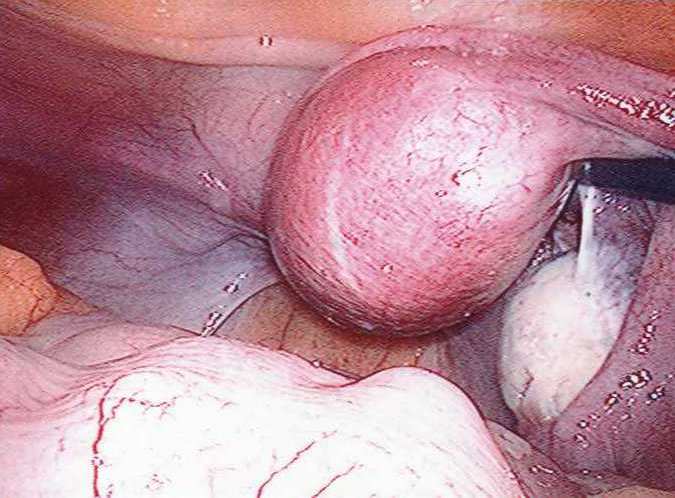

Supplement: Supplementary file 1 — Additional file 1: Figure S1: Laparoscopic image of uterus and right ovary. Note the absence of left ovary, left ovarian ligament and left fallopian tube. (JPEG 255 KB) [file 40064_2013_622_MOESM1_ESM.jpeg]

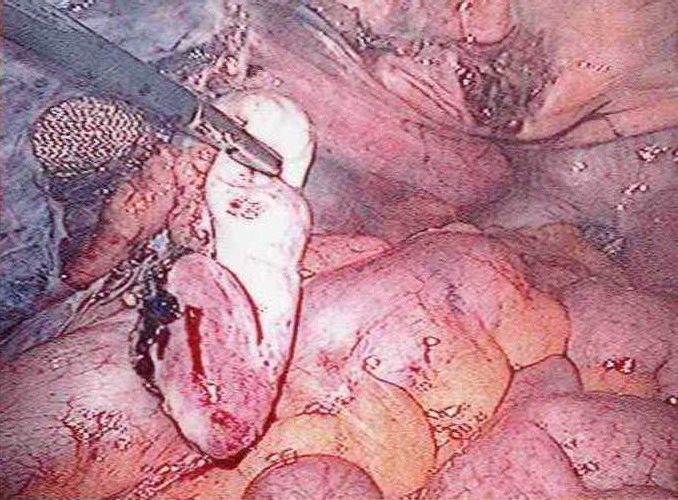

Supplement: Supplementary file 2 — Additional file 2: Figure S2: Laparoscopic image of left ovary following its removal from the inguinal canal and mesh repair of the hernia. (JPEG 329 KB) [file 40064_2013_622_MOESM2_ESM.jpeg]

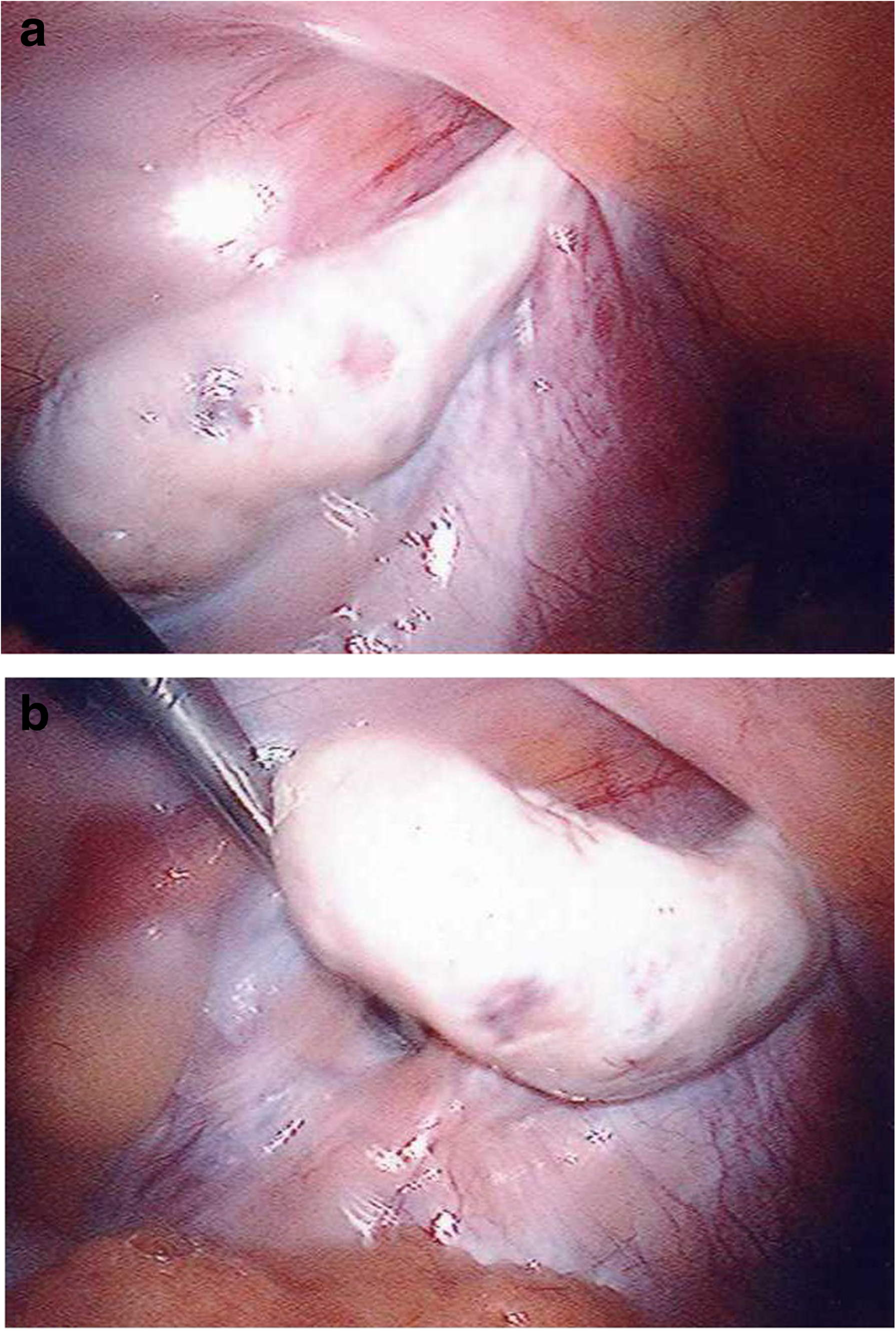

Supplement: Supplementary file 3 — Authors’ original file for figure 1 [file 40064_2013_622_MOESM3_ESM.tif]
